# The Crimean‐Congo haemorrhagic fever tick vector *Hyalomma marginatum* in the south of France: Modelling its distribution and determination of factors influencing its establishment in a newly invaded area

**DOI:** 10.1111/tbed.14578

**Published:** 2022-05-19

**Authors:** Madiou Thierno Bah, Vladimir Grosbois, Frédéric Stachurski, Facundo Muñoz, Maxime Duhayon, Ignace Rakotoarivony, Anaïs Appelgren, Clément Calloix, Liz Noguera, Théo Mouillaud, Charlotte Andary, Renaud Lancelot, Karine Huber, Claire Garros, Agnès Leblond, Laurence Vial

**Affiliations:** ^1^ CIRAD, UMR ASTRE Montpellier France; ^2^ ASTRE Univ Montpellier, CIRAD, INRAE Montpellier France; ^3^ Mas d'arboux Roquedur France; ^4^ Section of Epidemiology University of Zurich Zurich Switzerland; ^5^ VetAgro Sup INRAE, EPIA, Marcy L’étoile Lyon France

**Keywords:** correlative distribution modelling, France, *Hyalomma marginatum*, Tick

## Abstract

We developed a correlative model at high resolution for predicting the distribution of one of the main vectors of Crimean‐Congo haemorrhagic fever virus (CCHFV), *Hyalomma marginatum*, in a recently colonised area, namely southern France. About 931 *H. marginatum* adult ticks were sampled on horses from 2016 to 2019 and 2021 in 14 southern French departments, which resulted in the first *H. marginatum* detection map on a large portion of the national territory. Such updated presence/absence data, as well as the mean number of *H. marginatum* per examined animal (mean parasitic load) as a proxy of tick abundance, were correlated to multiple parameters describing the climate and habitats characterising each collection site, as well as movements of horses as possible factors influencing tick exposure. In southern France, *H. marginatum* was likely detected in areas characterised by year‐long warm temperatures and low precipitation, especially in summer and mostly concentrated in autumn, as well as moderate annual humidity, compared to other sampled areas. It confirms that even in newly invaded areas this tick remains exclusively Mediterranean and cannot expand outside this climatic range. Regarding the environment, a predominance of open natural habitats, such as sclerophyllous vegetated and sparsely vegetated areas, were also identified as a favourable factor, in opposition to urban or peri‐urban and humid habitats, such as continuous urban areas and inland marshes, respectively, which were revealed to be unsuitable. Based on this model, we predicted the areas currently suitable for the establishment of the tick *H. marginatum* in the South of France, with relatively good accuracy using internal (AUC = 0.66) and external validation methods (AUC = 0.76 and 0.83). Concerning tick abundance, some correlative relationships were similar to the occurrence model, as well as the type of horse movements being highlighted as an important factor explaining mean parasitic load. However, the limitations of estimating and modelling *H. marginatum* abundance in a correlative model are discussed.

## 1 INTRODUCTION

Zoonotic tick‐borne diseases transmitted by ticks of the Ixodidae family are an increasing health threat in Europe. Among tick‐borne zoonoses in Southern Europe, Crimean‐Congo haemorrhagic fever (CCHF) is one of the most concerning because of the severity of its clinical signs and high fatality rate in humans, as well as its potential to cause secondary nosocomial cases and even outbreaks. For these reasons, it is becoming a public health problem in Europe. While cases have been reported throughout the Balkans and the Anatolian Peninsula since the 1950s (Bakir et al., 2005; Christova et al., 2009; Papa et al., 2008), the south‐western regions of Russia recorded the disease only in 1999 after 27 years without any human cases (Onishchenko & Efremenko, 2004), and more recently, 9 autochthonous cases have occurred in Spain in 2013, 2016, 2018, 2020, and 2021 (Negredo et al, 2017; ECDC Cases of Crimean–Congo haemorrhagic fever in the EU/EEA, 2013–present). In these regions, the tick *Hyalomma marginatum* is considered to be one of the main vectors of the CCHF virus, as its wide geographical range is closely related to CCHF case distribution (Ergönul, 2006), except in Spain where it seems likely to be *Hyalomma lusitanicum*. Indeed, the CCHF virus was much more frequently detected in *H. lusitanicum*, whereas the infection rate of *H. marginatum* remained very low in the same region (Anabel Negredo et al., 2017; Ana Negredo et al., 2019; Estrada‐Pena et al., 2012; Moraga‐Fernández et al., 2021).

Since climate, especially temperature and humidity, is one of the main factors impacting hard ticks’ species distribution range (Cumming, 2002), there is mounting evidence that the emergence of tick‐borne diseases in Europe is linked to climate change (Andreassen et al., 2012; Daniel et al., 2003; El‐Sayed & Kamel, 2020; Tokarevich et al., 2011). To some extent, warmer conditions may prolong the active season of ticks and enhance the survival of non‐parasitic stages (i.e., eggs, questing stages, detached moulting stages, ovipositing females) in the environment. In some cases, this allows the establishment of tick colonies in new areas that are becoming more suitable for tick survival and development (Gray et al., 2009). Assessing the geographical extent of viable populations of the main tick vectors of pathogens is essential to assess the risk of emergence or re‐emergence of tick‐borne diseases. This can be determined using Hutchinson's ecological niche concept (1957), defined as a hypervolume shaped by the environmental conditions under which a species can persist indefinitely. Modelling approaches combining the characterisation of the ecological niche of a species with georeferenced information on easily measurable environmental variables allow drawing inferences on the distribution range of that species without systematic and updated sampling, thereby facilitating the identification of priority areas for surveillance. This can be done through process‐based models (Dormann et al., 2012; Kearney & Porter, 2009; Peterson et al., 2015), which use physiological information about the species in question, obtained from life‐history trait monitoring in controlled conditions that determine the range of environmental conditions in which the species can thrive. Another approach relies on correlative models (Guisan & Zimmermann, 2000) that directly relate environmental variables and species occurrence or abundance data, to identify major reliable parameters for species distribution and general equations for predictions. For the latter, species distribution models (SDMs) usually rely on presence‐only data obtained from museums, personal collections, published literature or citizen records, since both presence and absence data are hard to collect, update and validate. Presence‐only data are known to be prone to sampling biases because sighting distribution may only reflect highly visited regions, whereas presence–absence data sampled via systematic surveys are collected in both favourable and unfavourable regions without distinction (Elith et al., 2006; Fithian et al., 2015; Newbold, 2010).

Several modelling studies conducted at the European level to explain and predict the distribution of *H. marginatum* have emphasised that its most suitable habitats are characterised by high temperatures with low precipitation and low relative humidity (Estrada‐Peña & Venzal, 2007; Estrada‐Peña et al., 2011, 2012). Apart from climatic conditions influencing the development rate and activity of non‐parasitic stages, *H. marginatum* also needs the presence of vertebrate hosts to complete the blood meals required to moult from one development stage to the next. *Hyalomma marginatum* is a ditropic tick meaning that it is a two‐host species. The larvae and nymph stages feed on the same host species, which are small vertebrates such as lagomorphs, birds, hedgehogs and rodents, whereas adult stages usually feed on large ungulates such as horses, cattle, sheep, goat, deer or wild boar, and occasionally humans (Apanaskevich, 2004). As *H. marginatum* seems to be a generalist species, establishment may not be limited by host presence, as seems to be the case for other tick species. Although some host preference has been reported amongst its wide range of hosts (Grech‐Angelini et al., 2016), they should be considered with caution as patterns can differ from one region to another (Mccoy et al., 2013). Likewise, preferred local host density may likely influence the dynamics and abundance of tick populations rather than their establishment. In addition, incorporating host availability in predictive models is challenging because host density data are often unavailable or not detailed at a local geographical scale, especially for wild hosts and even more so for small vertebrates. However, for some wild species such as wild boar or rodents, it has been demonstrated that vegetation could be a relevant proxy for predicting abundance since it reflects the availability of food resources (Pittiglio et al., 2018). Vegetation can also directly influence the survival of non‐parasitic tick stages, especially by reducing the risk of desiccation under dry and hot meteorological conditions. For example, only one model that was developed for *H. marginatum* used normalised difference vegetation index (NDVI) as a proxy for vegetation hydric stress to differentiate densely forested areas and open areas with low or inexistent chlorophyllic activity (Estrada‐Peña & Venzal, 2007). However, NDVI is not able to differentiate between more precise vegetation types (e.g., natural vs agricultural zones among open areas), nor characterise global landscape. Therefore, predictions resulting from this model are globally relevant but may lack local accuracy. Finally, another study tried to cross‐tabulate tick presence reports with landcover data but resulted in counterintuitive associations, as *H. marginatum* presence was associated with urban, agricultural and forested areas (Estrada‐Peña et al., 2016).

In France, *H. marginatum* has been reported on the island of Corsica as early as 1946 (Delpy, 1946; Morel, 1959). This tick is now abundant and widely distributed all over the island (Grech‐Angelini et al., 2016). On the mainland, rare historical records date from 1940 to the 1970s (Morel, 1959; Rageau, 1972). Most of these records were not able to confirm the presence of *H. marginatum* due to doubtful identification, absence of information concerning specimen's development stage, or because they seemed to likely correspond to a punctual introduction by migratory birds of immature stages that successfully moult as adults without any subsequent tick population establishment. The presence of viable and abundant *H. marginatum* populations in continental France was only recently reported (Vial et al., 2016), suggesting that the south of France may represent a newly colonised, suitable area for *H. marginatum* for which the extent of the invasion is still unknown.

Based on tick sampling carried out from 2016 to 2019 and 2021, this study provides an updated geographical distribution map for *H. marginatum* in southern continental France. This presence and absence data, along with the mean number of ticks per examined animal as a first estimation of abundance, were used to develop a statistical model that aims to give new insights into the environmental factors limiting or facilitating the permanent establishment of *H. marginatum* populations, at a finer spatial resolution than previous ecological niche models and using multiple parameters to describe precise climate and habitat factors. Regarding biotic and abiotic conditions, this study provides a robust model for predicting suitable areas in southern France that may be colonised by this tick vector.

## 2 MATERIALS AND METHODS

### 2.1 Study area and time of sampling

We chose to sample ticks in equestrian structures because they are most likely to be infested in areas where *H. marginatum* is established, as horses seem to show higher infestation rates by *H. marginatum* adult stages than any other domestic ungulates (Grech‐Angelini et al., 2016; Santos‐Silva et al., 2011). By equestrian structure, we refer to any facility that houses horses; this definition includes stables for horse owners, leisure structures offering occasional horse riding, equestrian centres that provide lessons and usually participate in competitions, horse‐breeding structures and traditional structures rearing horses used in taurine training.

We sampled structures across 14 French continental departments forming a link on the Mediterranean coast between the borders of Spain and Italy, which are two countries where *H. marginatum* is known to be present. A department corresponds to a NUTS 3 (Nomenclature of Territorial Units for Statistics) administrative division. Some of the selected departments have a Mediterranean environment, while others are characterised by a transitional zone where climate tends to become much cooler and humid and is assumed to be less suitable for tick establishment (Joly et al., 2010).

We first conducted tick collections in 2016, in the department of Bouche‐du‐Rhône (Desjardins et al., 2018); subsequent samplings dedicated specifically to detecting *H. marginatum* were then carried out in 2017, 2018, 2019 and 2021. The sample from 2021 was only used for model validation as it occurred after the analysis. To ensure data homogeneity, all tick samplings were conducted in spring (from April to July, mainly in May), which is the peak period of activity regarding the *H. marginatum* adult stage.

### 2.2 Tick sampling and identification

We aimed to sample ticks on at least eight horses per structure and if possible, from horses using different pastures to have a good representation of infestation in the structure. If the structures had fewer than eight horses, we sampled all available individuals and considered their infestation status as representative of the visited structure if they have access to the whole environment around the structure.

Tick collection consisted of a thorough full body search for any ticks on the animal. When feeding on a host, *H. marginatum* is usually attached around the genitals and anus, between the thighs, between the udders, and on the abdomen, in rare instances. While exploring its host, prior to attachment at the definitive engorgement site, the tick usually waits behind the pasterns or near the hooves (Apanaskevich, 2004). On horses, other tick species can be found attached on dorsal side of the host, or near the mane or tail. All ticks were removed manually and placed in plastic tubes. Each evening, ticks were sorted by sex and developmental stage, and the species was determined according to its morphological characteristics using relevant species descriptions and identification keys (Apanaskevich & Horak, 2008; Estrada‐Peña et al, 2004; Claudine, 2007). After identification, ticks were frozen in liquid nitrogen. Once returning from the field, ticks were stored at −80°C in the laboratory for later use.

### 2.3 Equestrian structures selection

We included structures that used as little acaricide treatment as possible and which provided horses with access to vegetated areas (Tirosh‐Levy et al., 2018) to favour horses’ exposure to the environment and therefore to ticks. A structure was included in our sample once we obtained owner consent. Furthermore, before our visit, we specifically asked owners not to apply any parasite treatment and, upon visiting, we verified the absence of such treatment (acaricides, but also anthelminthic ivermectin that may have an effect on ticks). Horses that had recently received treatment (less than 14 days) were excluded from the study. During the visit, GPS coordinates of each horse structure or pasture (when far from the main structure) were recorded. In addition, we filled out a form compiling information about the structure (owner, activity, nature and number of animals), the management of horses (last movements, last treatments, any unusual observations), and the scope of the visit (number of horses examined, number of horses parasitised by ticks, and specifically by *H. marginatum*). Since horses’ movement within their respective areas should directly impact the number of encounters with ticks and therefore infestation rate and probability of presence, this information was used to determine horse‐movement classes, categorised as followes: (1) Limited (level‐1): horses staying in their paddocks and rarely going on strolls for a day or less; (2) Medium (level‐2): horses trekking for a few days, but staying within the study zone; (3) Important (level‐3): Horses going out of the study zone for competitions, breeding or trade.

We reported in each structure the mean number of ticks found on examined horses. We assumed that the tick detection error on an infested horse was negligible since the tick attachment location on horses is well known for *H. marginatum*. In addition, most of the examinations were conducted jointly by two observers. We considered that *H. marginatum* was absent from a structure when no tick was found on a sufficient or representative number of horses, and after ensuring the absence of acaricide treatments; if treatments were conducted within the last 14 days, absence was validated if some specimens from other tick species were found on horses. We considered that the tick was established as a permanent population in a structure when at least two ticks were found on one or two horses, provided that they had not recently moved. For *H. marginatum*, a single tick found on a horse may be due to an engorged nymph having been introduced by a migratory bird (Capek et al., 2014; Černỳ & Balát, 1957; Jameson et al., 2012), then moulting locally into an adult and finally attaching to a horse, without indicating a local development cycle. This type of single detection has already been reported in Germany, the United Kingdom and Sweden (Chitimia‐Dobler et al., 2019; Grandi et al., 2020; Hansford et al., 2019; Lesiczka et al., 2022). Therefore, when a single *H. marginatum* was found in the whole structure, it was labelled as a “single presence” and was not considered to be sufficient reliable evidence of *H. marginatum* establishment (i.e., the existence of a permanent population in the area) to be used in the analysis. As female ticks engorge for 7–12 days and males can remain attached for at least a month, an observed infestation could also result from a recent horse movement from already‐infested areas. Collected information regarding recent horse movements helped to detect such doubtful detection cases, and only tick count data from horses that did not move from another department or country during the month prior to sampling were considered.

### 2.4 Environmental data collection and treatment

We used a combination of CORINE Land Cover (CORINE Land Cover 2018, Version 2020_20u1) and BD forêt (BD Forêt® version 2.0) to characterise the habitats. CORINE Land Cover (CLC) is a biophysical inventory of land use according to a 44‐item nomenclature. This inventory was produced at a 1/100,000 scale by visual interpretation of satellite images in 39 European countries and was updated in 2018. Pixels are 100‐m wide (area of 10,000 m^2^). The BD Forêt (BDF) version 2.0 is a reference database for forested areas and semi‐natural environments, which is compiled and made available by the *Institut National de l'Information Géographique et Forestière* (IGN) (French Institution of Geographic and Forest Information). It describes the forest and natural plant formations by a land cover approach in pixels wider than 70 m (areas larger than 5,000 m^2^). It is elaborated by visual interpretation of infrared images and is delivered according to a departmental segmentation, with updates ranging from 2014 to 2019. In addition, BDF contains information on a finer scale than CLC. For these reasons, we were much more confident in using BDF despite being limited to forested areas. Therefore, we first used CLC and then transferred BDF values to CLC in overlapping pixels that were classified as forests using the packages “raster” (Hijmans, 2020) and “rgdal” (Bivand et al., 2020). This resulted in a composite raster with a pixel resolution of 100^*^100 m. From this composite raster and for each data point, we extracted land cover proportions within a radius of 5 km around the GPS coordinates to have information on the environment around the equestrian structures, which likely reflects the environment where horses can become infested by *H. marginatum*. Among the several kilometre radii that we tested within the 1 to 10 km range, we chose 5 km as a sensible trade‐off between bias and variance, which produced the best area under the receiver operating characteristic curve (AUC) using the leave‐one‐out method. AUC is a threshold‐independent measure of a model's ability to discriminate between sites where a species is present, versus sites where it is absent (Hanley & McNeil, 1982). To avoid model overfitting, the resulting 76 land‐cover classes from BDF and CLC fusion were reclassified into 7 generic classes: Humid areas such as inland marshes and rice fields, open natural areas such as sclerophyllous vegetation and sparsely vegetated areas, open forests, broad‐leaved forests, coniferous forests, urban or peri‐urban areas and agricultural areas. These classes were chosen as they reflected the main categories of habitats encountered within the study region, with a distinct capacity to retain humidity and allow for sun exposure, as well as a diverse availability of hosts. For each sampling point, the resulting seven variables represent the relative proportion of each habitat in a 5‐km radius. Regression with such compositional explanatory variables, which sum to unity, can be misleading due to their non‐Euclidean geometry (Aitchison 1982), which causes invalid estimation of the regression parameters of the model inference. Therefore, via the package “compositions” (Van den Boogaart et al., 2020), we used an *isometric log‐ratio (ILR) transformation* (Hron et al., 2012), which maps the original domain into a 6‐dimensional Euclidean space. The resulting variables, called *balance coordinates*, represent the coefficients of the original data with respect to an orthonormal Euclidean basis on the transformed space.

Regarding climate, we used the SAFRAN (*Système d'Analyse Fournissant des Renseignements Adaptés à la Nivologie*) analysis module of the French National Meteorological Service, from 2000 to 2018. SAFRAN provides both ground and altitude weather estimates from a numerical interpolation model fitted to weather measurements from the French network of meteorological stations. Weather estimates are presented in a regular 8‐km grid covering France. To characterise the climatic conditions of each sampling point, we collected monthly means of daily maximal and minimal temperatures, precipitation, potential evapotranspiration and relative humidity that are common features for describing climate, averaged across the 18 selected years to remove inter‐annual variation and reflect the climate of the last decades coinciding with the timeframe when the establishment of *H. marginatum* may have occurred. Principal component analysis (PCA) was performed on climate data to reduce dimensionality and avoid multicollinearity in our regression model. PCA allows for the description of a complex dataset using a small number of uncorrelated variables called principal components (PCs) that retain most of the variability and ecological meanings from the original data. For defining PCs, climatic data were extracted from each grid cell within the extent of the selected prediction area (south of France from the coastline to the latitude of 45°N), using the packages “ade4” (Dray & Dufour, 2007), “sf” (Pebesma, 2018) and “factoextra” (Kassambara & Mundt, 2020). Then, for each tick sampling point, PC coordinates were extracted from the cell where the point was located, using the package “rgeos” (Bivand & Rundel, 2020).

### 2.5 Statistical analysis

Out of the 169 sampling points used to develop the model (data from 2021 excluded), 113 had no ticks and 13 were single observations, corresponding to about two‐thirds of count observations being zeros. Thus, a zero‐inflated Poisson mixture (ZIP) model (Lambert, 1992) was used to estimate the probability of occurrence and abundance of *H. marginatum*. The ZIP model assumes that the data are generated by two underlying processes, which results in two distinct layers in the model: The “zero layer” and the “count layer”. The first process is modelled by a Bernoulli model, where the probability *π*_i_ of tick absence, resulting in an observed outcome of 0, may depend upon the covariates. The second process models the abundance of ticks, conditional to their presence, as a Poisson regression of mean *μ*_i._ The response variable was the number of *H. marginatum* collected in each sampling point with the number of horses examined as an offset. The explanatory variables were the linear and quadratic functions of the three principal components of the climatic variables, the ILR balance coordinates of habitat and the level of horse mobility. Quadratic functions of principal components of climatic variables were included as it was hypothesised that the relationship between tick abundance and climate was not linear, with an expected optimum beyond which the population density will decline (Estrada‐Peña et al., 2011; Ogden et al., 2004; Randolph, 1997).

Model selection was based on Akaike's information criteria (AIC) and likelihood ratio tests (LRTs). The first model included all predictors in both the Binomial and Poisson components; we then conducted a backward stepwise elimination process to select the most predictive variables in the final model (Akaike, 1974; Woolf, 1957). At each step, we compared the model with and without each variable and removed the variable for which the LRT test was non‐significant and for which the AIC value was the lowest. The selection stopped when for all remaining variables, the LRT test was significant and the difference between AIC values was lower than 2. The resulting model had the lowest value of AIC and best goodness of fit.

Using the most parsimonious model to interpret the effect of each habitat under ILR transformation on the probability of presence and abundance of *H. marginatum*, we fitted multiple, equivalent but differently parametrised ZIP models such as the first balance coordinate representing one habitat type over the geometric means of all others as it is easily interpretable.

To assess the potential geographical expansion of *H. marginatum* in all of southern France, we then drew a raster map of the predicted probability of occurrence (presence/absence) based on the coefficients of the selected model, with a resolution of 100^*^100 m. To do this, we sampled all the points within the prediction zone such as the resulting raster drawn from these points have a resolution of 100^*^100 m and then we extracted climate and habitat data for each point. The predictive performance of the model was evaluated using three data sources: (i) The training dataset using the “leave one out” method, which consists of successively fitting the model without one observation and testing the prediction of the model at the removed observation; (ii) the collection of testimonies by people made aware of the *H. marginatum* existence thanks to several reports in the media (only testimonies confirmed with photos and with precise locations were included). As these data were presence‐only, and given that we made a buffer of 5 km around the sampling points, we sub‐divided the study area into 10‐km grids and took the centroid of these grids as a pseudo‐absence point so that each point was spaced 5 km apart and that there was no overlap between their buffer. (iii) The presence–absence data obtained from the last 2021 field survey, which were not included in the training dataset. We used AUC to assess the concordance between presence–absence records and the model predictions. For statistical analyses, we used the R statistical software R version 4.0.2 with the packages MASS (Venables & Ripley, 2002), lmtest (Zeiles & Hothom, 2002) and pscl. (Jackman et al., 2007)

## 3 RESULTS

### 3.1 *H. marginatum* distribution in the south of France

Out of 2588 ticks collected from horses among the whole study area, the most frequently identified species were *Rhipicephalus bursa* with 1147 individuals (44%), including 572 males, 557 females and 18 nymphs, followed by *H. marginatum* with 931 (36%) individuals, including 524 males and 407 females. The other tick species consisted of 244 *Ixodes ricinus*, including 18 males, 173 females and 53 nymphs (9%), 97 *Dermacentor marginatus*, including 47 males and 50 females (4%), 69 *Dermacentor reticulatus*, including 30 males and 39 females (3%), 63 *Haemaphysalis punctata*, including 24 males, 33 females and 6 nymphs (3%), and 37 *Rhipicephalus* of the *sanguineus sensu lato (s.l.)* group, including 13 males and 24 females (1%). Where *H. marginatum* was present, it was predominant (75%) with 931 individuals among the 1234 collected ticks in the sampling points.

A total of 188 equestrian structures, i.e., sampling points, were visited. The sampling points covered the Mediterranean eco‐climatic zone as well as surrounding climates but their distribution was uneven, with a few sampling sites in some departments at the border of the study area, such as Aveyron, Lozère and Ariège, while the other departments were sampled on at least 6 sampling points. An average of 10 horses per equestrian structure was examined (ranging from 2 to 35). Horses from 82 of these structures stayed in their paddock and rarely went for a stroll (level‐1 movement), animals from 59 structures were kept in pastures or moved regularly for a few days (level‐2) and horses of the 47 remaining structures participate in competition or breeding events (level‐3).

**FIGURE 1:**
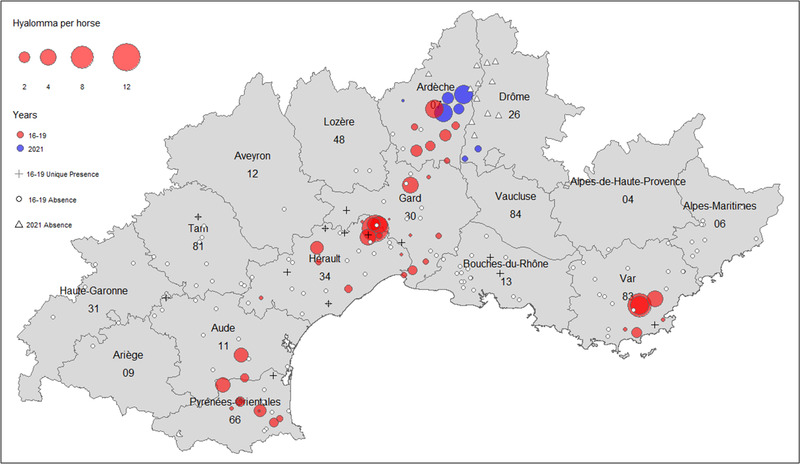
Localisation of the sampling sites of *Hyalomma marginatum* across 14 French departments. Coloured circles represent the sites where *H. marginatum* is present and circle size corresponds to the average number of *H. marginatum* per horse in each equestrian structure. Red circles correspond to the surveys conducted from 2016 to 2019, while blue circles correspond to the 2021 survey. White circles correspond to sites where *H. marginatum* was considered absent during the 2016–2019 surveys and white triangles for the 2021 survey. Black crosses represent the sites where only one specimen of *H. marginatum* was found during the 2016–2019 surveys (not included in the modelling)

In total, 63 of the 188 visited structures were infested by *H. marginatum*, including 13 single presences, while the remaining 125 were considered negative. The mean parasitic load by *H. marginatum* (mean number of *H. marginatum* per examined horse) ranged from 0 to 11.43, depending on the structure. The tick was reported in 7 of the 14 sampled departments, but its distribution was not homogeneous over this zone. There were “clusters” of presence around Perpignan in Pyrénées‐Orientales, in Corbières hills in the south of Aude, in the hinterland of Montpellier in Hérault, in the north of Gard, the southern part of Ardèche and the extreme south of Drôme, as well as around the Maures mountainous massif in Var (Figure 1). Absences were mainly reported from Alpes‐Maritimes, Drôme and Bouches‐du‐Rhône to the east, and in the western part of the sampling zone. Although single presences were mainly located around presence clusters, a few were reported in areas presenting only absences such as in Bouches‐du‐Rhône, Aude and Tarn.

### 3.2 Correlations between climatic variables

The first three axes of PCA accounted for 87.7% of the variability of climatic data. They were therefore all included in the model.

**FIGURE 2:**
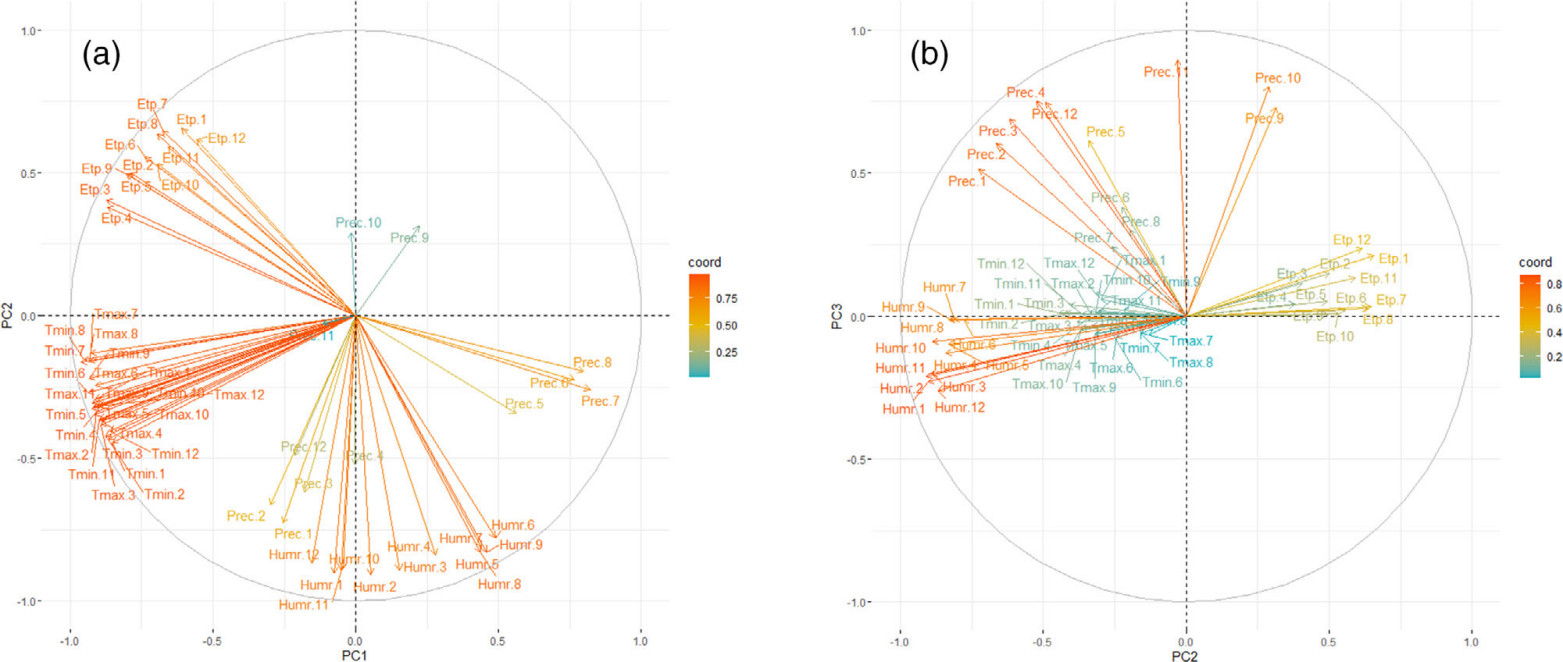
Principal component analysis of the climatic data, under the principal components 1 (PC1) and 2 (PC2) (2A) and the principal components 2 (PC2) and 3 (PC3) (2B). Colours represent the squared sum of the coordinates of the climatic variables on the principal components. Red corresponds to high correlation whereas blue means low correlation. The horizontal axis corresponds to PC1 in Figure 2a and PC2 in Figure 2b, and the vertical axis to PC2 in Figure 2a and PC3 in Figure 2b. *T*max: maximum temperature, *T*min: minimum temperature, ETP: potential evapotranspiration, Prec: precipitation, Humr: relative humidity. The numerical suffix corresponds to the month (1 for January to 12 for December)

The first principal component (PC1) accounted for 50.1% of the variability, with minimal and maximal monthly mean temperature scores strongly and negatively correlated with PC1 (scores > 0.86; Figure 2A). Similarly, monthly mean potential evapotranspiration scores had a moderate‐to‐strong negative correlation with PC1 (scores = 0.55–0.78; PC1, Figure 2A). Precipitation during summer (June to August) was correlated heavily and positively with PC1 (scores > 0.76; PC1, Figure 2A). Relative humidity during spring and summer (May to August) had a moderate and positive correlation with PC1 (scores = 0.43–0.49; PC1, Figure 2A). PC1 thus discriminates climatic conditions that are hot all year round (high temperature and potential evapotranspiration) and dry during summer (low precipitation and relative humidity) from climatic conditions that are cooler all year round and wetter during summer. PC1 can be interpreted as a temperature and summer precipitation index.

The second principal component (PC2) explained 28.7% of the variability, with monthly mean relative humidity negatively and strongly correlated with PC2 (scores > 0.77; PC2, Figure 2B), as well as a similar but moderate relationship with winter precipitation (January to March) (scores 0.61–0.72; PC2, Figure 2B) and a positive moderate relationship with potential evapotranspiration over the year (scores 0.38–0.65; PC2, Figure 2B). PC2 thus discriminates climatic conditions that are relatively dry from climatic conditions that are more wet. PC2 can thus be interpreted as a humidity index.

The third principal component (PC3) explained 8.9% of the variability and was mostly defined by precipitation during autumn (September to November), with scores strongly and positively correlated with PC3 (scores ≥ 0.73; PC3, Figure 2B). PC3 can thus be interpreted as an autumn precipitation index.

### 3.3 Habitats sampling effort

We measured *H. marginatum* abundance on structures in seven different habitats. Their distribution in our sample according to various criteria is presented in Table 1.

**TABLE 1:** Sampling effort of each class of habitat. Habitat: Class of habitat

Habitat	*n* sampled	*n* pres sampled	*n* most sampled	*n* pres most sampled	*n* abs most sampled	*n* upres most sampled
Humid	59	14	13	1	12	0
Open natural	143	41	27	14	9	4
Open forest	155	41	0	0	0	0
Coniferous forest	157	41	36	8	27	1
Urban or peri‐urban	151	41	2	0	2	0
Agricultural	169	43	84	18	60	6
Broad‐leaved forest	162	43	7	2	3	2

*Note*: *n* sampled: number of times the habitat was present in the 5 km buffer around all sampling points; npres sampled: number of times the habitat was sampled in the 5 km buffer around the sampled presence points; *n* most sampled: number of times the habitat was dominant; *n* pres most sampled: number of times the habitat was dominant for presences; *n* abs most sampled: number of times the habitat was dominant for absences; *n* upres most sampled: number of times the habitat was dominant for single presences.

### 3.4 Effects of climate and habitat on *H. marginatum* distribution

**FIGURE 3:**
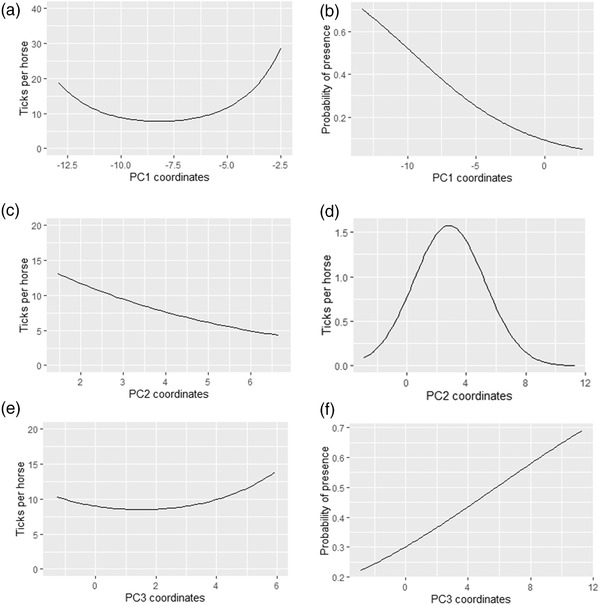
Conditional effects of climate synthetic variables PC1, PC2 and PC3 on the conditional tick abundance (3A, 3C, 3E) and the probability of tick presence (3B, 3D, 3F) using the most parsimonious model, when all other parameters are fixed to their mean. The *X*‐axis on conditional tick abundance curves (left side) represents the range of PCs values on observed tick presence

The results of the ZIP model are presented in Table 2 and Figure 3. Although the “zero layer” of the model estimates the effect of explanatory variables on the probability of absence, results will be interpreted in terms of probability of presence by switching the sign of the estimates. The terms that remained in the most parsimonious model after the backward selection for the “zero layer” of the model were the three principal components, the squared PC2, and the ILR balance coordinates of habitats. Most of the terms were retained for the “count layer”, except for the squared PC2.

**TABLE 2:** Results of the zero‐inflated Poisson model with the best AIC and LRT tests, with estimate values, standard error and p‐value for each parameter in both model layers. Habitat coefficients correspond to the first ILR coordinates where the proportion of the considered habitat is the first compositional part. Grey coloured grids correspond to parameters that were not included in the most parsimonious model

		Count layer			Zero layer		
		Estimate	Standard error	p‐value	Estimate	Standard error	p‐value
Intercept		2.14	0.46	**0.00** ***	4.49	1.13	**0.00** ***
PC1		0.66	0.12	**0.00** ***	0.23	0.14	**0.09** *
PC1^2		0.04	0.008	**0.00** ***			
PC2		−0.22	0.08	**0.00** ***	−0.69	0.46	0.14
PC2^2					0.10	0.06	**0.08** *
PC3		−0.08	0.06	0.2	−0.11	0.12	0.34
PC3^2		0.02	0.007	**0.00** ***			
Movement	Level‐1 (ref)						
	Level‐2	0.32	0.1	**0.00** ***			
	Level‐3	−0.77	0.16	**0.00** ***			
Habitats (ILR)	Agricultural	0.16	0.07	**0.01** **	−0.17	0.24	0.49
	Urban or peri‐urban	0.17	0.05	**0.00** ***	0.43	0.20	**0.04** **
	Open forest	−0.57	0.06	**0.00** ***	0.14	0.22	0.53
	Broad‐leaved forest	0.16	0.09	0.09	0.18	0.28	0.52
	Coniferous forest	−0.21	0.09	**0.02** **	−0.31	0.26	0.23
	Humid	−0.32	0.05	**0.00** ***	0.22	0.13	**0.10** *
	Open natural	0.61	0.07	**0.00** ***	−0.37	0.21	**0.08** *

*** <0.001;

** <0.05;

* <0.1.

PC1 had a significant negative effect on the probability of presence of *H. marginatum* but showed a significant positive effect on the parasitic load (Table 2; Figures 3A and 3B). This means that *H. marginatum* is likely present in areas with climatic conditions that are relatively hot all year round and dry during the summer (Figures 1, 2 and 3 in supplementary data). Regarding the parasitic load, over the range of PC1 values on observed presence, the relationship is convex. With increasing PC1 values, a decrease in the predicted parasitic load is observed until a minimum is reached, followed by an exponential increase that rapidly exceeds the infestation rates observed on horses and thus does not reflect the reality. We noticed that the range of PC1 values on the observed presences are exclusively negative, thus corresponding to hot and dry conditions in summer. Considering the plots drawn from the model when covariates values are fixed to their mean, a 50% probability of presence is reached as soon as the mean annual temperature is 16.3°C and mean monthly summer precipitation is below 32 mm. For a maximal parasitic load of 12 ticks/horse as observed in the field, mean annual temperature remains above 17°C and mean monthly summer precipitation below 32 mm.

**FIGURE 4:**
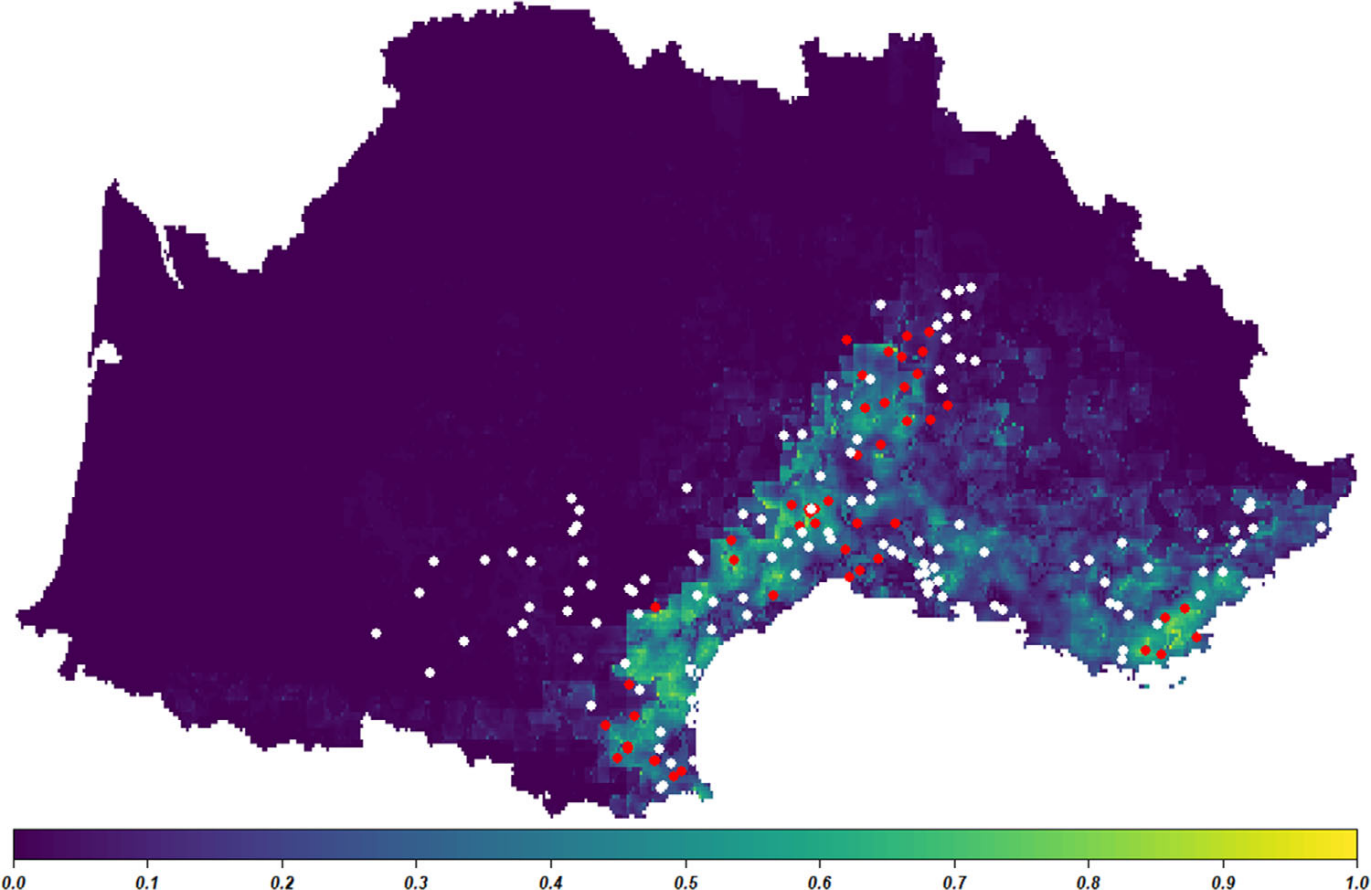
Predicted probability of presence for *H. marginatum* in the south of France, using the most parsimonious model (dark blue: null probability to bright yellow: highest probability). Red circles represent the observed presence and white circles observed absences from testimonies and surveys

PC2 had a negative effect on the parasitic load and a positive effect on the probability of tick presence up to a point where it becomes negative (Table 2; Figure 3D). This means that *H. marginatum* is likely present under fairly dry conditions (Figure 4 and 5 in supplementary data) until a certain threshold after which conditions seem to become too dry. Conversely, increasing humidity is favourable to tick abundance. Nevertheless, for tick abundance, the range of PC2 values on the observed presences is exclusively positive, corresponding to dry conditions only. Maximal probability of presence is predicted for 71% mean annual relative humidity and mean monthly winter precipitation of 51 mm. An effective maximal parasitic load of 12 ticks/horse is obtained under a relative humidity of 73% and a mean monthly winter precipitation of 57 mm.

PC3 had a positive effect on the probability of tick presence. On tick parasitic load, in the observed presence range of PC3's values, the relationship is slightly convex. (Figures 3E and 3F). However, the relationship is nearly flat suggesting little involvement of PC3 on tick parasitic load. Thus, *H. marginatum* is likely to be present in areas presenting high precipitation in autumn (Figure 6 in supplementary data). A 50% probability of presence is reached as soon as mean monthly autumn precipitation is above 187 mm.

Open natural habitats had a significant positive effect on the probability of *H. marginatum* presence whereas urban or peri‐urban and humid habitats had significant negative effects, meaning that this tick is likely to be present in non‐flood‐prone open natural areas where human activities are scarce. Regarding the “count layer” of the model, most habitat parameters had significant effects on the tick parasitic load. In the same manner, as for probability of tick presence, open natural areas were favourable to large tick populations and humid habitats were unsuitable. Open and coniferous forests were considered unsuitable while higher proportions of agricultural and urban or peri‐urban habitats had a positive effect on the number of ticks found on horses. Finally, horse movement only had a significant effect on tick abundance. Horses are more likely to be exposed to a larger amount of ticks when strolling around or trekking for a few days within the sampling zone (level‐2) than horses staying in their pastures and paddocks (level‐1), but also more than horses that are used to traveling far away for competitions, breeding or trade (level‐3).

### 3.5 Prediction of *H. marginatum* distribution and model validation

Figure 4 shows the predicted probability of *H. marginatum* presence in southern France. Its presence was predicted only in coastal areas along the Mediterranean Sea with a small incursion towards the north along the Rhône valley. Within this zone, the highest probabilities were localised on the western margin from Pyrénées‐Orientales to Ardèche while areas to the east of the Rhône River, except Var, were considered less suitable for the establishment of *H. marginatum*. A certain heterogeneity was also observed within the suitable zone, with areas such as Camargue, Etang de Berre in Bouches‐du‐Rhône, the plains of Béziers, Perpignan in Hérault and Pyrénée‐Orientales, respectively, which were predicted as unsuitable by the model and sometimes confirmed by observed absences. Outside this zone, the western part of the country is predicted to be unsuitable once reaching the plains of Aude, Tarn and Haute‐Garonne, as well as the mountainous areas of Aveyron, Lozère, northern Gard and Ardèche. In the eastern part, the limit is less clear in Drôme, Vaucluse, Bouches‐du‐Rhône and Alpes‐de‐Haute‐Provence. While all observed absences of *H. marginatum* were well predicted by the model in the western part of the study area, some minor discrepancies between predictions and observations were observed, with suitability overestimation at the eastern margin of Camargue and the hinterland of Var, as well as suitability underestimation in the northern part of Ardèche. This resulted in moderate efficiency of the model using the “leave one out” validation method (AUC of 0.658 in ROC analysis). However, results improved using the testimonies and the occurrences of the 2021 survey for model validation (AUC of 0.761 and 0.833, respectively).

## 4 DISCUSSION

In this study, we built a correlative model for predicting the distribution of *H. marginatum*, namely in the South of France, which has recently been colonised by this tick species, using updated presence and absence data collected from the field. We also measured the mean parasitic load of horses as a proxy of tick relative abundance. We associated these field data with climatic and habitat variables to better understand the environmental factors that limit or facilitate the establishment and the proliferation of this invasive tick. Based on this model, we were able to predict suitable areas for the current establishment of *H. marginatum* on a larger zone than sampled in the south of France.

Our sampling data showed that *H. marginatum* is already established in several departments in southern France, from Pyrénées‐Orientales in the west to Var in the east, and Ardèche in the north. The fact that tick presence is spatially aggregated in three clusters centred on locations with particularly high tick abundances, and that several single‐presence recordings have been documented around these clusters suggest a possible ongoing colonisation process of contiguous‐free favourable habitats stemming from presence clusters of already‐established tick populations due to host movements (Lockwood et al., 2005). However, some other single‐presence recordings were reported outside such zones in areas where *H. marginatum* is absent. Considering the ability of *H. marginatum* immature stages to feed on birds and the suitability of these natural locations (Camargue in Bouches‐du‐Rhône, Gô in Tarn, Lauragais in Aude) to welcome migratory birds in spring, this may correspond to punctual long‐distance introductions of nymphs that are able to moult after detaching from birds and parasitise horses at the adult stage. However, environmental conditions in these zones may remain unsuitable to allow *H. marginatum* to successfully complete its full development cycle and for the subsequent establishment of a viable population. Possible short‐ and long‐distance tick diffusion through vertebrate host movements raises questions for possible spatial autocorrelation between presence data. Although results were not shown in the present paper, we tested for spatial autocorrelation in the residuals of our model, using Moran test, and it was not significant.

Using presence/absence data, the “zero layer” of our model demonstrated that climatic conditions of sampling points that are warmer all year round and sufficiently dry during summer, yet have high precipitation in autumn and moderate‐to‐low year‐round relative humidity within the range of sampled values, favour the presence of *H. marginatum*. Climate effects are supported by previous correlative analyses where *H. marginatum* presence or infestation were positively correlated to low rainfall and high temperatures (Estrada‐Peña et al., 2007; Tirosh‐Levy et al., 2018) and where extreme humidity was unsuitable for self‐sustained populations of ticks (Estrada‐Peña et al., 2011). Morel (2003) described *H. marginatum* as a thermophilic and xerophilous tick. He reported that *H. marginatum* successfully develops and produces self‐sustained populations at a mean temperature in July above 24°C. However, low temperatures do not appear to affect the survival of *H. marginatum* in laboratory conditions and viable adults have been recorded at temperatures below −20°C in Russia (Estrada‐Peña et al., 2011; Hoogstraal, 1979). We support the hypothesis that temperature has a direct effect on critical development processes of certain stages, namely egg incubation and nymph moulting in spring and summer, as ticks need to moult into adults (i.e., an overwintering stage that sufficiently resists to low temperature) before winter (Estrada‐Peña et al., 2011; Gray et al., 2009; Kotti et al., 2001; ECDC Hyalomma marginatum ‐ Factsheet for experts). Conversely, we assume that the observed positive correlation with low summer but high autumn precipitations likely reflects the dependency of *H. marginatum* on the Mediterranean and transitionary Mediterranean climates, which are both characterised by specific rainfall patterns in addition to high annual temperatures (Joly et al., 2010). However, larvae of *H. marginatum* that emerge in spring or summer must show high resistance to desiccation, namely by finding sufficiently humid microhabitats to resist. In the future, it would be interesting to measure the direct effect of these different climatic variables on the moulting of nymphs in natural conditions using close monitoring of multiple cohorts under diverse meteorological conditions. Regarding the presence of viable *H. marginatum* populations under specific climatic conditions (in contrast to single‐presence data, indicating possible introduction events within a larger geographic zone in southern France), we assume that *H. marginatum* does not have sufficient ecological plasticity to establish itself outside the Mediterranean climate. As proposed by Estrada‐Peña (2008), such a crucial aspect of phenotypic adaptation that is rarely explored in ticks should be further investigated.

Regarding the effects of habitat on tick presence, in agreement with previous observations (Akimov & Nebogatkin, 2011; Hoogstraal, 1979; Uspensky, 2002), we demonstrated a positive relationship of *H. marginatum* presence with open, natural habitats such as scrubland, a xerophytic biotope commonly found in a non‐uniform fashion in southern France under the Mediterranean climate. Such natural habitats are assumed to provide suitable vegetation (grass and herbs in spring, wood products such as leaves in summer and oak acorns in autumn) for the presence of wild hosts of *H. marginatum* such as lagomorphs, birds and wild boar. In addition, these habitats still offer sufficient protective vegetation against desiccation to non‐parasitic stages. Conversely, our model showed that humid habitats are detrimental to the presence of *H. marginatum*, although these habitats have a high diversity of potential vertebrate hosts (birds, lagomorphs, rodents, horses, cattle and so on) for both immature and adult stages that may be encountered. This relationship could be explained by the xerophilic nature of this tick with the assumed inability of non‐parasitic stages to survive in flooded areas, as egg mortality and development anomaly at high RH had been reported (Buczek, 2000; Morel, 2003). This confirms the findings of Fernández‐Ruiz and Estrada‐Peña (2021) who reported that a decrease in soil humidity within a Mediterranean‐type habitat was associated with an increase in environmental suitability for *H. marginatum*. Such humid habitats are commonly found in Camargue, which settles at the mouth of the Rhône River in Bouches‐du‐Rhône (13), where *H. marginatum* introductions are very likely through migratory birds (as shown by several single‐presence records) but where large plains remain humid all year long, favouring cattle and horse rearing but not tick establishment. Concerning urban or peri‐urban habitats, they have a negative effect on tick establishment, which could be easily explained by the absence of wild animal hosts and domestic ungulates for *H. marginatum*, as well as the lack of vegetation cover for non‐parasitic stages. Albeit, some studies have even suggested permanent populations of certain tick species in cities (Hansford et al., 2017; Kowalec et al., 2017; Klemola et al., 2019; Sormunen et al., 2020). To our knowledge, no such observation has been reported for *H. marginatum*. This may be due to the fact that available hosts in urban settings are usually not the preferred hosts of this tick species, with the exception of wild boar, but colonisation of suburbs by such ungulates is often the result of overabundance in adjacent areas (Warren, 2011; Ciach and Fröhlich, 2019). Wild boar populations are abundant in the south of France (Alexander et al., 2016) but precise information on their distribution is still unavailable to assess the trophic preferences of *H. marginatum* regarding this ungulate in the region. Concerning open forests that were primarily assumed to be possible suitable habitats for *H. marginatum* establishment, our model did not show a significant effect concerning presence probability. Although the majority of habitats were well represented in all our sampling points (Table 1), the open forest habitat was never predominant and has a limited surface in our study area, making it more complicated to estimate its effect. Furthermore, we correlated relative habitat proportion surrounding each sampling point to the occurrence and abundance of *H. marginatum*; however, it is most likely that one habitat may become more suitable in combination with others, or that the assembling of different types of habitat into landscapes may also be important. This aspect should be further investigated.

Our predictive map of *H. marginatum* presence probability in the south of France using the “zero layer” of our most parsimonious model allowed for a precise delimitation of the current expansion of the tick. As discussed earlier, the tick's current suitable predicted areas are restricted to zones with a typical or transitionary Mediterranean climate. Within this general zone, some reported incongruences between predictions and observations contributed to decreasing model efficiency but may reflect a complex and ongoing invasion process. The apparent model overestimations at the margins of Camargue and the hinterlands of Var and Alpes‐Maritimes deserve particular attention, as their climatic conditions may not be included in the fundamental ecological niche of this tick species, or they may currently be free areas but could become invaded in the following years. This process could also explain the differences in prediction power between the internal validation and the external ones. Indeed, the predictive performance of our model was moderate with the “leave‐one‐out method” since some observed absences collected from 2016 to 2019, and used for calibrating the model, may be suitable but falsely negative because they have not yet been invaded by *H. marginatum*. Conversely, the efficiency of our model increased with the other validation methods using testimonies and the 2021 sampling survey, as they were based on much more recent reports. However, as testimonies are presence‐only, pseudo‐absences were created on the whole prediction zone that is larger than the Mediterranean sampling zone, and thus artificially increases the extent and the number of well‐predicted presence and the AUC scores (Lobo et al., 2008). Nevertheless, such bias does not exist in the 2021 sampling survey for which we obtained the best AUC scores, as we decided to target areas where the tick was assumed to be absent to better assess its northern margin of distribution. This reinforces the ability of our model to predict absences and that the pseudo‐absences sampled outside of the Mediterranean sampling zone were likely to be true absences. However, the existence of other unmeasured explanatory variables may also explain discrepancies and should be further investigated.

Measured mean parasitic loads as proxies of tick abundances used for the “count layer” of the model are disputable and need to be considered with caution. Indeed, if *H. marginatum* is still in a colonizing phase, populations may not be in equilibrium. A sampling point with higher abundance does not necessarily mean that the climate and the habitat are much more suitable; this can also be due to a longer period of establishment of the population, allowing for more individuals to be generated. Indeed, as well described for some invasive tick species such as *Rhipicephalus microplus* (De Clercq et al., 2013), we assumed that secondary colonisation by *H. marginatum* from one or several original established populations is a homogeneous process where the tick will colonise the surrounding favourable habitats in a spreading pattern via the movement of wild animals, particularly ungulates and resident birds. In addition, in this study, we estimated the abundance of *H. marginatum* by measuring the mean number of ticks per examined horse. This was based on the hypothesis that, at the peak of the adult activity, most ticks are searching for large ungulates to engorge, and that examined horses can sample a large part of the targeted area. However, horses’ parasitic loads also vary according to husbandry practices in each visited structure, as it was clearly shown in our model through the significant effect of horse movements on tick abundance. Many more *H. marginatum* were found on horses that were trekking or staying in a pasture for a few days, which corresponds to the best occasion for horses to be exposed to ticks, rather than horses that stay in their paddock or go outside of the study zone for competitions, breeding or trade. Interestingly, in a previous study, pastures were reported as the main factor influencing exposure to *H. marginatum* (Tirosh‐Levy et al., 2018). Although horses cover long distances when going to competitions, they do not necessarily travel into infested areas; they are instead housed in stables or sparse paddocks during their displacement and are often treated against parasites. Rotation in pastures is also an important practice that may modify horse exposure to ticks. In one of the sampling zones included in the study where the infestation is regularly monitored, this rate was shown to re‐increase in summer as soon as a new pasture was offered to horses (Stachursky, personal communication); However, for the rest of our sampling points, which were only visited once, it was difficult to assess such possible bias. Another potential source of bias is caused by undisclosed grooming practices, which can lead to an underestimation of tick abundance. Indeed, as *H. marginatum* adults remain attached to hosts for an average of 10 days (Morel, 2003), measures of mean parasitic load reflect the number of ticks attached during this period, unless horse owners are used to regularly detaching ticks. This was not always mentioned during visits with owners, especially in pensions where horses may belong to different owners. Nevertheless, we believe that it did not affect our results on presence detection since a total absence of ticks (all species included) was observed in only 9 of the sampling points, which lacked information regarding treatment or grooming practices.

Considering all these limitations on the “count layer” of the model, we must be cautious about the interpretation of abundance results. It was however noticeable that open natural habitats were favourable for tick abundance, similarly with tick presence. Regarding climatic conditions, even though relationships seemed different to those observed for tick presence, the range of climatic conditions was similar. Within warm and dry climatic conditions that are suitable for tick presence, increasing humidity might be slightly favourable to tick abundance as suggested by the relationships of PC1 and PC2. These results are in line with the common xerophilic nature of the tick that is present only in dry and hot Mediterranean areas, provided that residual humidity is available either directly in the environment or within sheltered microhabitats, for the survivability of non‐parasitic stages. A more reliable abundance model would require dedicated sampling to better estimate the infestation rates under an extended range of climatic conditions.

## CONFLICT OF INTEREST

The authors declare that they have no competing interests.

## ETHICS STATEMENT

The authors confirm that the ethical policies of the journal, as noted on the journal's author guidelines page, have been adhered to and the appropriate ethical review committee approval has been received. The CIRAD code of ethics guidelines were followed.

## Supporting information

Figure 1. Sampled mean temperature range, in red the distribution for observed presence.
Figure 2. Sampled mean spring and summer relative humidity range, in red the distribution for observed presence.
Figure 3. Sampled mean potential evapotranspiration range, in red the distribution for observed presence.
Figure 4. Sampled mean summer precipitations range, in red the distribution for observed presence
Figure 5. Sampled mean winter precipitations range, in red the distribution for observed presence.
Figure 6. Sampled mean autumn precipitations range, in red the distribution for observed presence.

## Data Availability

The data that support the findings of this study are available from the corresponding author upon reasonable request.
