# Supplementary material for: The Crimean‐Congo haemorrhagic fever tick vector Hyalomma marginatum in the south of France: Modelling its distribution and determination of factors influencing its establishment in a newly invaded area
Source: Transbound Emerg Dis. 2022 May 19;69(5):e2351–65. doi: 10.1111/tbed.14578 (PMC9790221; doi:10.1111/tbed.14578)
Supplement: Supplementary file 1 — Figure 1. Sampled mean temperature range, in red the distribution for observed presence. Figure 2. Sampled mean spring and summer relative humidity range, in red the distribution for observed presence. Figure 3. Sampled mean potential evapotranspiration range, in red the distribution for observed presence. Figure 4. Sampled mean summer precipitations range, in red the distribution for observed presence Figure 5. Sampled mean winter precipitations range, in red the distribution for observed presence. Figure 6. Sampled mean autumn precipitations range, in red the distribution for observed presence. [file TBED-69-e2351-s001.docx]

**Supplementary material**

**
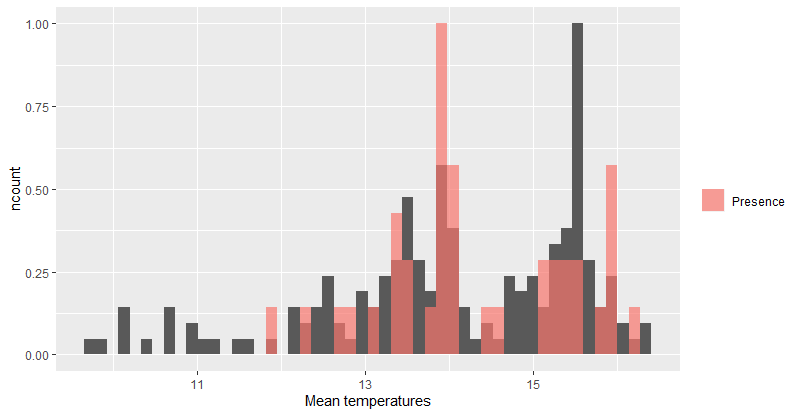
**

Figure 1. Sampled mean temperature range, in red the distribution for observed presence.


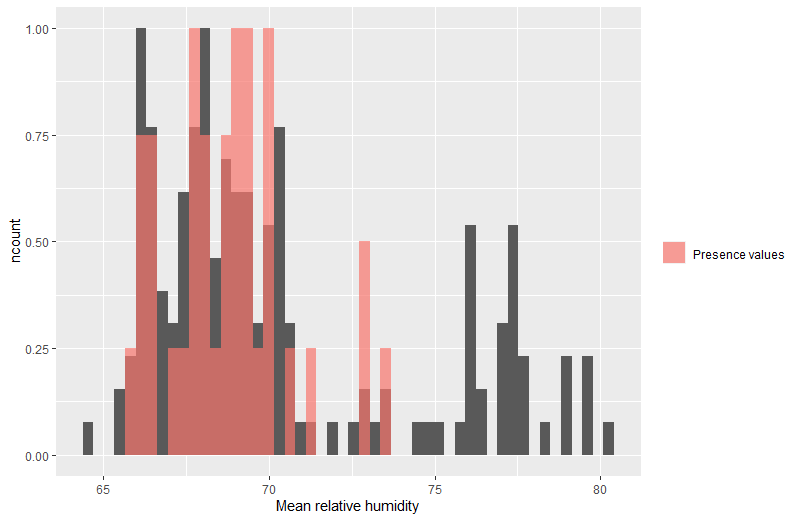


Figure 2. Sampled mean spring and summer relative humidity range, in red the distribution for observed presence.

**
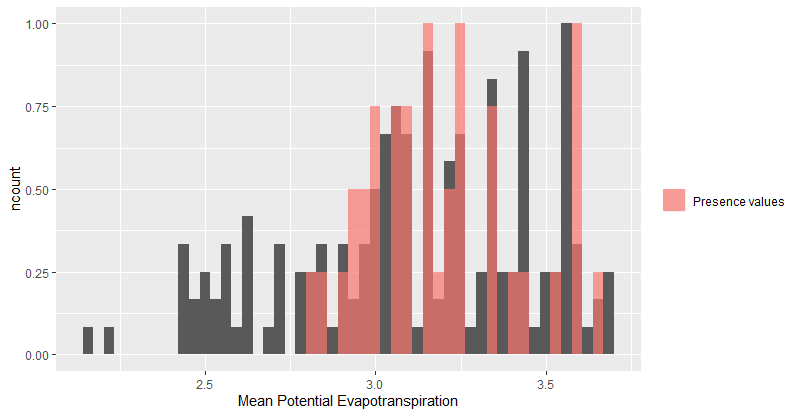
**

Figure 3. Sampled mean potential evapotranspiration range, in red the distribution for observed presence.

**
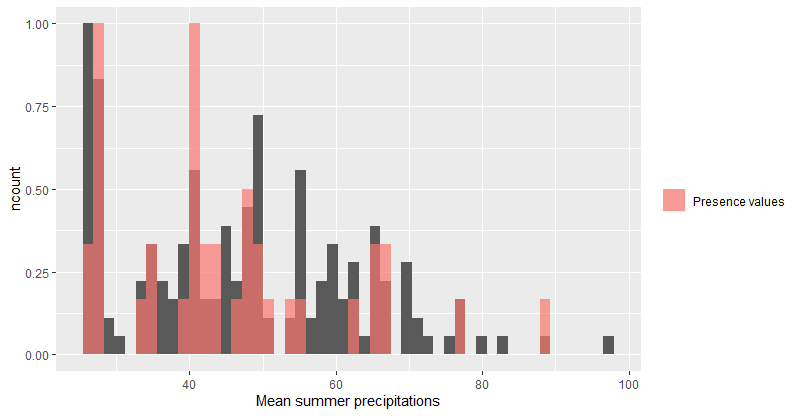
**

Figure 4. Sampled mean summer precipitations range, in red the distribution for observed presence

**
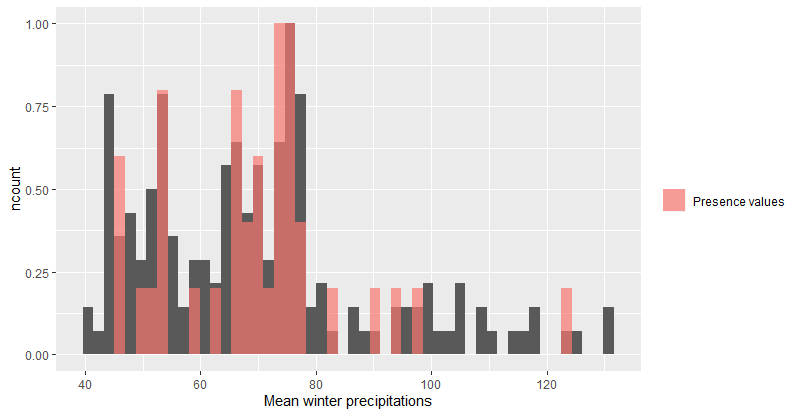
**

Figure 5. Sampled mean winter precipitations range, in red the distribution for observed presence.

**
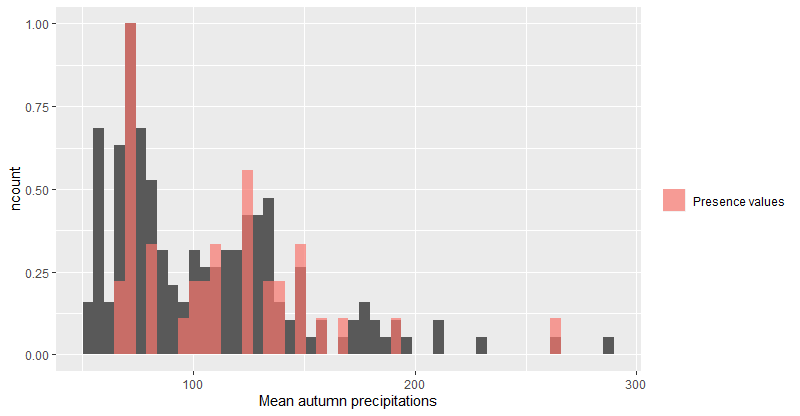
**

Figure 6. Sampled mean autumn precipitations range, in red the distribution for observed presence.
